# IL-1b-Bearing NETs: Bridging Inflammation to Early Cirrhosis in Hepatitis B

**DOI:** 10.3390/ijms26125733

**Published:** 2025-06-15

**Authors:** Maria Ntinopoulou, Theocharis Konstantinidis, Anna Chalkidou, Eleni Papagianni, Aikaterini Skeva, Maria Panopoulou, Akrivi Chrysanthopoulou

**Affiliations:** 1 Laboratory of Molecular Immunology, Department of Biological Applications & Technology, Faculty of Health Sciences, University of Ioannina, 45332 Ioannina, Greeceeleni.papagianni2000@gmail.com (E.P.); 2 Laboratory of Microbiology, Department of Medicine, Faculty of Health Sciences, Democritus University of Thrace, 68100 Alexandroupolis, Greece; theoxari_ko@yahoo.gr (T.K.);; 3 First Department of Internal Medicine, University General Hospital of Alexandroupolis, 68100 Alexadroupolis, Greece; annachal22@gmail.com

**Keywords:** hepatitis B virus (HBV), neutrophils, neutrophil extracellular traps, inflammation, interleukin (IL)-1b, liver fibrosis, cirrhosis

## Abstract

Hepatitis B virus (HBV) infection is one of the most dangerous viral diseases, with innate immunity representing the first line of defense against the virus. In this branch of the immune system, neutrophils are considered key cellular mediators. To better understand the implication of neutrophils in the distinct stages of the disease, HBV-infected patients were enrolled in this study and categorized into three groups: patients with acute infection, chronic infection under treatment, and at early cirrhotic stage. To elucidate the role of inflammatory mediators and cellular mechanisms of neutrophilic origin in the course of the infection, both ex vivo and in vitro studies were performed. Increased levels of C-C motif chemokine ligand 2 (CCL2), interleukin (IL)-18, IL-33, and citrullinated histone H3 (CitH3)—an accurate marker of neutrophil extracellular traps (NETs)—were detected in the circulation of patients with acute infection or early cirrhosis. In parallel, sera from the aforementioned patient groups induced the formation of IL-1b-bearing NETs in neutrophils from healthy individuals. These inflammatory NETs affected primary fibroblasts towards acquiring a pro-fibrotic phenotype. These results suggest that NETs could be regarded as mediators in hepatitis B manifestations, while their therapeutic targeting could enhance the management of early-stage cirrhotic patients.

## 1. Introduction

Hepatitis is an inflammatory response of the liver that can either progress to liver fibrosis, cirrhosis, or cancer or, under certain conditions, be reversible [1,2,3,4,5]. Among the five major hepatitis viruses, hepatitis B virus (HBV) is considered one of the most infectious and dangerous [6]. Despite the existence of vaccines and antiviral drugs that attempt to control the new infections [6], HBV is still a global health problem with significant morbidity and mortality potential.

HBV infection is a cause of both acute and chronic hepatitis. Upon exposure to HBV, the individual’s immune system attempts to mount strong responses to eliminate infected cells and produce neutralizing antibodies. Otherwise, acute hepatitis can progress to chronic infection, placing patients at significant risk of liver disease. In addition, clinical remission of both chronic infection and acute hepatitis is possible. It can be achieved either spontaneously or after therapeutic intervention; however, this does not mean complete eradication of the virus [7,8,9].

During infection, virus components interact with distinct immune cells, guiding various cellular mechanisms and immune pathways. Briefly, HBV components can activate host cell pattern recognition receptors (PRRs) and induce the expression of type I interferon as well as the secretion of pro-inflammatory mediators [10,11]. Interestingly, secreted proteins have a wide range of actions against the virus. Some of them mediate antiviral responses, while others augment the effects of HBV and result in chronic inflammation [12,13,14,15]. Among the most potent pro-inflammatory cytokines, involved in HBV infection, is interleukin (IL)-1b [12,16,17].

In addition, innate immune cells, such as neutrophils, have a key regulatory role in infectious conditions. Neutrophils are the predominant leukocytes in peripheral blood and the first cell population to approach sites of infection. They can recognize pathogen-associated molecular patterns (PAMPs) via Toll-like receptors (TLRs) [18,19,20,21,22] and, subsequently, they can exert antimicrobial effects through the mechanism of phagocytosis, degranulation, or neutrophil extracellular trap (NET) release [19,20].

It should be clarified that NETs can exert pleiotropic functions that go beyond pathogen clearance. Depending on the disease microenvironment, neutrophils and, consequently, NETs can adapt phenotypically and functionally [23,24,25,26] promoting mechanisms of inflammation, thrombosis, fibrosis, or cancer [11,21,22,23,25,26,27,28,29]. Indicatively, the interaction of NETs with mesenchymal-derived cells, such as fibroblasts, plays a crucial role in enhancing fibrotic manifestations [25,30,31].

While the role of neutrophils/NETs in infections is well-recognized, the studies that have investigated the involvement of neutrophils in HBV disease are limited. More specifically, one study has argued that polymorphonuclear cells from asymptomatic carriers of hepatitis B are enriched in HBV DNA [32]. There are also data that have demonstrated the ability of recombinant hepatitis B e antigen (HBeAg) to influence neutrophil migration and the production of reactive oxygen species [33]. Furthermore, findings have shown that activated neutrophils, overexpressing the CD68 surface marker, programmed cell death ligand 1 (PD-L1), and IL-8, are found in the periphery of treatment-naïve chronic HBV patients [34].

In summary, significant experimental data on HBV have been provided in recent years. However, the involvement of innate immune mechanisms in the disease requires further investigation. Consequently, the present study associates the mechanism of NETs with specific diseases stages. At the same time, it examines the phenotype of NETs, i.e., their protein load, as well as their ability to influence the inflammatory and early fibrotic aspects of the disease. Finally, it provides data suggesting that therapeutic targeting of NETs could limit fibrotic liver damage triggered by HBV.

## 2. Results

### 2.1. Inflammatory Proteins and NETs Are Detected in the Circulation of HBV Patients

**Figure 1:**
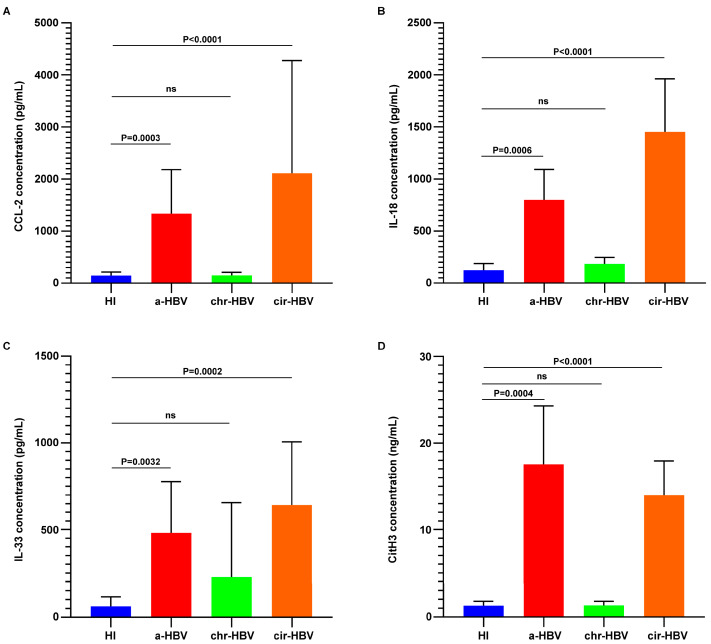
Inflammatory proteins and neutrophil extracellular traps (NETs) are present in the circulation of a-HBV and cir-HBV patients. Levels of (**A**) C-C motif chemokine ligand 2 (CCL2), (**B**) interleukin(IL)-18, and (**C**) interleukin(IL)-33 in the serum of HBV patients (*n* = 11 subjects per group). (**D**) Concentration of citrullinated histone H3 (CitH3), representing NET release, in the serum of HBV patients (*n* = 11 subjects per group). For (**A**–**D**), data are shown as mean ± SD, Kruskal–Wallis test, followed by Dunn’s multiple comparison test. All conditions were compared to HI. Statistically significant: *p* < 0.05. ns: non-statistically significant. HI: healthy individuals; a-HBV: acute HBV; chr-HBV: chronic HBV infection under treatment; cir-HBV: early cirrhotic stage.

To assess the inflammatory milieu of HBV infection, blood sera derived from distinct phases of the infection were analyzed by a bead-based multiplex immunoassay. We observed that C-C motif chemokine ligand 2 (CCL2), interleukin (IL)-18, and interleukin (IL)-33 were elevated in the circulation of patients with either acute infection (a-HBV) or early cirrhosis (cir-HBV), in contrast to healthy individuals (HI) (Figure 1A–C). On the other hand, the expression levels of these mediators in patients with chronic HBV (chr-HBV) were comparable to the control group (Figure 1A–C).

CCL-2, IL-18, and IL-33 are involved in the functional responses of human neutrophils [35,36,37,38,39], including NET formation [40,41,42]. Considering that NETs are a key neutrophil response mechanism, patient sera were then examined for the presence of circulating NET structures. Increased NETs were detected in the circulation of patients with acute infection (a-HBV) or early cirrhosis (cir-HBV), in contrast to controls, as assessed by a citrullinated histone H3 (CitH3) ELISA (Figure 1D). On the contrary, the presence of NETs was limited in samples from chronic patients (chr-HBV) and similar to control group (Figure 1D). These observations indicate that inflammatory responses tend to be increased in both the acute and early cirrhotic phases of the disease, as evidenced by the presence of inflammatory mediators and CitH3-positive structures in the periphery of patients. Moreover, a positive correlation between the HBV inflammatory burden and NETs is suggested (Figure S1).

### 2.2. The Inflammatory Milieu of HBV Infection Triggers the Formation of NETs In Vitro

**Figure 2:**
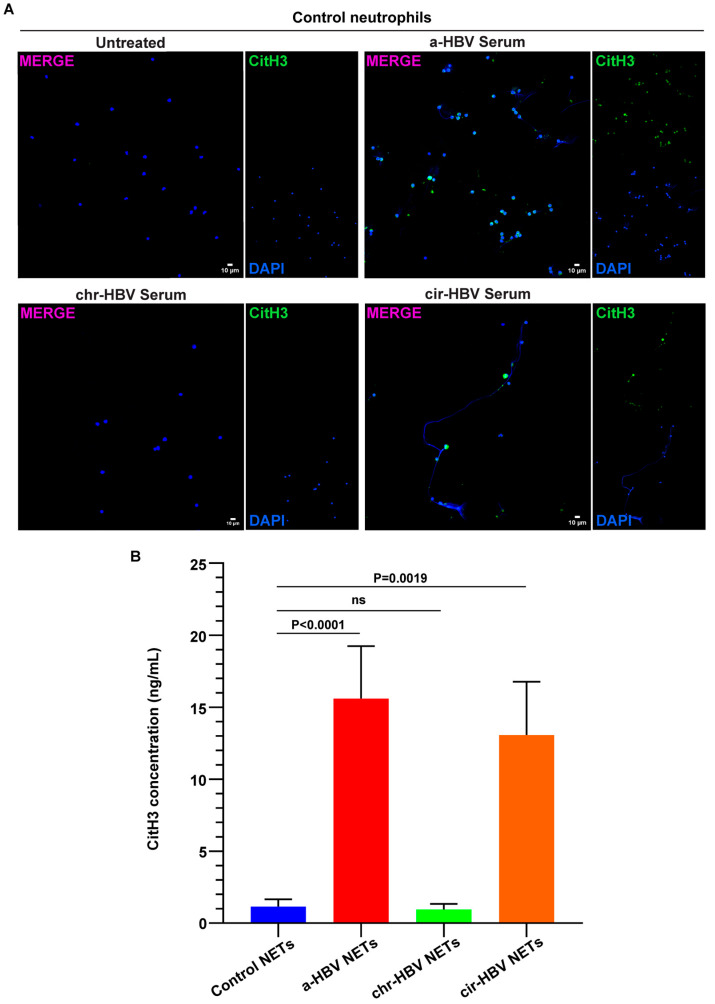
The inflammatory a-HBV and cir-HBV microenvironment induces NET formation in vitro. (**A**) Fluorescence confocal microscopy images showing citrullinated histone H3 (CitH3) staining, representing NETs (blue: DAPI; green: CitH3; original magnification: 400×). A representative example of 6 independent experiments is shown. (**B**) Levels of CitH3, representing NET release, after in vitro stimulation of control neutrophils with serum from HBV patients (*n* = 11). For (**B**), data are shown as mean ± SD, Friedman test, followed by Dunn’s multiple comparison test. For (**B**), all conditions were compared to control NETs. Statistically significant: *p* < 0.05. ns: non-statistically significant. a-HBV: acute HBV; chr-HBV: chronic HBV infection under treatment; cir-HBV: early cirrhotic stage.

To examine the potential influence of the disease microenvironment on neutrophil population, in vitro cell-based studies were performed. Accordingly, neutrophils were isolated from healthy individuals (control neutrophils) and stimulated with serum samples from patients with acute (a-HBV) or early cirrhotic (cir-HBV) disease. Under these culture conditions, control neutrophils formed NETs, as assessed by immunofluorescence (Figure 2A) and CitH3 ELISA on isolated NET structures (Figure 2B). On the other hand, sera from patients with chronic HBV (chr-HBV) were unable to drive neutrophils towards NET formation (Figure 2A,Β). Together, these findings support an association between the inflammatory microenvironment of HBV and NET formation.

### 2.3. NETs, Formed in Response to the Inflammatory Environment of HBV, Are Coated with IL-1b

**Figure 3:**
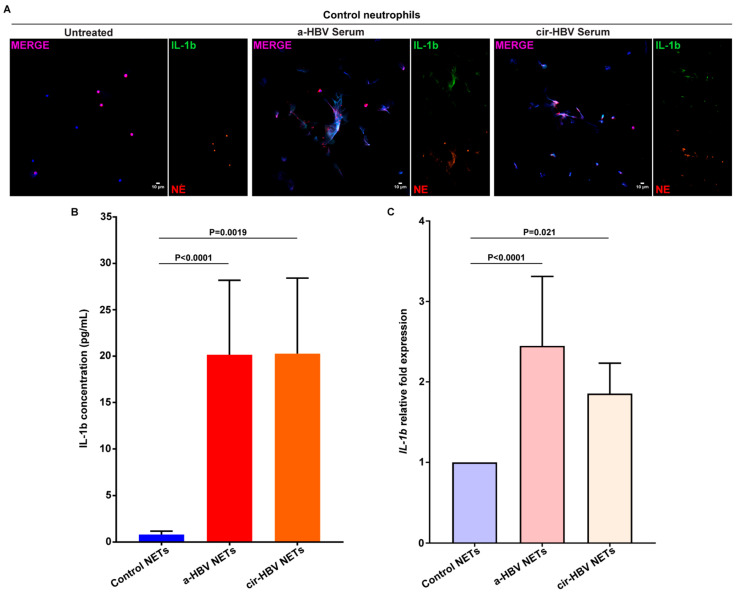
IL-1b-bearing NETs are formed in the presence of a-HBV or cir-HBV microenvironment in vitro. (**A**) Fluorescence confocal microscopy images showing neutrophil elastase (NE) and interleukin(IL)-1b staining (blue: DAPI; red: NE; green: IL-1b; original magnification: 400×). A representative example of 6 independent experiments is shown. (**B**) Concentration of IL-1b on in vitro isolated NETs, after stimulation of control neutrophils with serum from HBV patients (*n* = 11 independent experiments). (**C**) IL-1b expression in control neutrophils treated with HBV serum, as assessed by RT-qPCR (*n* = 6 independent experiments). For (**B**,**C**), data are shown as mean ± SD, Friedman test, followed by Dunn’s multiple comparison test. For (**B**,**C**), all conditions were compared to control NETs and untreated, respectively. Statistically significant: *p* < 0.05. a-HBV: acute HBV; cir-HBV: early cirrhotic stage.

A number of recent studies have shown that potent inflammatory proteins expressed in NET structures are disease-specific and can determine the phenotype of each disease [25,26,29,30,43]. Therefore, to investigate the protein composition of HBV NETs, control neutrophils were incubated with serum samples from patients with acute infection (a-HBV) or early cirrhosis (cir-HBV). In both conditions, control neutrophils formed NETs bearing mature IL-1b, as verified by immunofluorescence (Figure 3A) and IL-1b ELISA on isolated NET structures (Figure 3Β). Moreover, neutrophils stimulated with HBV sera showed intracellular overexpression of IL-1b, compared to unstimulated ones, as determined by real-time qPCR (Figure 3C). In summary, the disease environment leads to the formation of IL-1b-coated NETs, which could be involved in acute HBV responses as well as events associated with the early cirrhotic stages.

### 2.4. Inflammatory NETs Enhance the Fibrotic Dynamic of Mesenchymal Cells In Vitro

**Figure 4:**
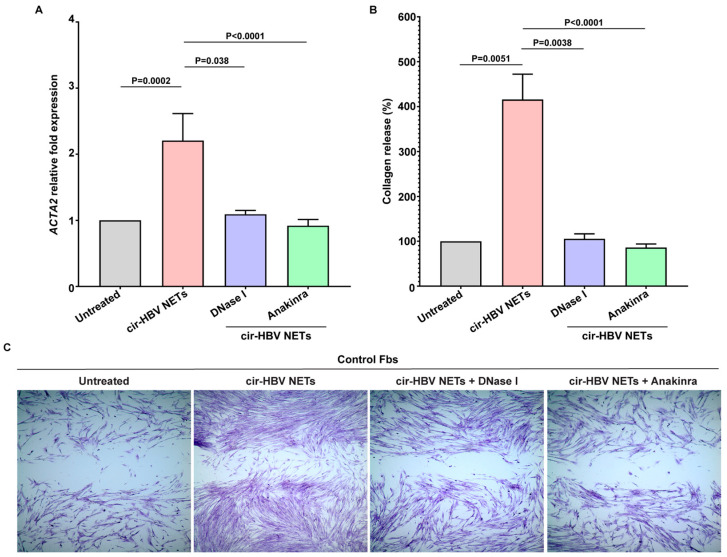
The migratory/wound healing dynamic and collagen production of fibroblasts are enhanced by cir-HBV NETs in vitro. (**A**) Smooth muscle actin alpha 2 (ACTA2) expression (*n* = 6 independent experiments), (**B**) collagen production (*n* = 6 independent experiments), and (**C**) migratory/wound healing capacity of control fibroblasts (Fbs) upon stimulation with in vitro isolated cir-HBV NETs (original magnification: 40×). For (**C**), a representative example of 6 independent experiments is shown. In all experiments, NET structures were dismantled with DNase I and interleukin(IL)-1b signaling was blocked in Fbs using a recombinant human IL-1 receptor antagonist (Anakinra). For (**A**,**B**), data are shown as mean ± SD, Friedman test, followed by Dunn’s multiple comparison test. For (**A**,**B**), all conditions were compared to untreated. Statistically significant: *p* < 0.05. cir-HBV: early cirrhotic stage.

There are strong indications that infectious as well as inflammatory factors can lead to harmful activation of fibroblasts and this in turn to fibrotic tissue damage [25,26,30,44,45,46,47]. In the same context, fibroblast immune responses can be influenced by cell-to-cell communication mechanisms, including interactions between fibroblasts (Fbs) and neutrophils via NETs [25,30,31,48,49]. Hence, to study the early cirrhotic stage of the disease, a co-culture system between HBV NETs and human Fbs was established. Under these culture conditions, cells were highly activated compared to unstimulated Fbs, as evidenced by the overexpression of smooth muscle actin alpha 2 (ACTA2) (Figure 4A). Fbs also acquired a potent pro-fibrotic phenotype, as verified by the increased collagen production (Figure 4B) and enhanced migratory/wound healing capacity (Figure 4C).

In contrast, disassembly of NETs with DNase Ι abolished the pro-fibrotic potential of Fbs, as confirmed by the marked reduction in ACTA2, collagen secretion, and migration/wound healing capacity (Figure 4A–C). A similar effect was observed upon blocking IL-1b signaling by applying a recombinant human IL-1 receptor antagonist (Anakinra) to Fbs (Figure 4A–C). Collectively, these results support an interplay between HBV NETs and Fbs that could possibly contribute to HBV-associated liver cirrhosis.

## 3. Discussion

Our manuscript provides data supporting NET formation as a characteristic feature of the various stages of HBV. NET structures, detected in individuals with acute hepatitis B as well as in individuals with chronic infection accompanied by early liver fibrosis, appear to shape the course of the disease.

In cases of infection, chemokines have a crucial role; they can enhance the recruitment of immune cells to the sites of inflammation and contribute to the elimination of pathogens [50,51]. Specifically, CCL2 represents an activator of macrophages, monocytes, and neutrophils, driving events of uncontrolled inflammation and fibrosis [52,53,54]. Similarly, IL-18, a member of the IL-1 cytokine superfamily, activates neutrophils, contributing to the early phases of inflammatory and innate immune responses [55,56,57,58]. Circulating IL-18 levels are elevated in several inflammatory conditions [59,60], while data have also linked the cytokine to liver fibrosis [61]. In addition, IL-33, a chromatin-associated nuclear cytokine from the IL-1 superfamily, is released upon cellular damage and exerts potent actions upon infection [62]. In liver diseases, IL-33 directly affects innate lymphoid cells as well as hepatic stellate cells (HSCs) that produce collagen [63,64]. Our data, obtained from a bead-based immunoassay, are consistent with these observations. They show that CCL2, IL-18, and IL-33 are involved in distinct stages of the disease and, particularly, in the onset of the disease, as well as in the hepatic fibrosis that may occur during the chronicity. Therefore, we can suggest that inflammatory events as well as fibrotic manifestations of HBV could be influenced by the aforementioned secreted proteins.

Neutrophils upon contact with pathogens or under the influence of numerous inflammatory stimuli can form NETs, which can function either beneficially or detrimentally during an immune response [65]. Concerning HBV, data has suggested that viral proteins can affect the formation of NETs in patients with chronic infection [66]. On the other hand, there are findings supporting that NET structures are more abundant in HBV-positive patients with hepatocellular carcinoma (HCC) compared to HBV-negative HCC patients [67]. Here, we demonstrate that both the acute phase of the disease and the chronic phase with early cirrhosis are characterized by the profuse presence of circulating NETs. Furthermore, we show that the disease microenvironment, which is enriched in CCL2, IL-18, and IL-33, can reproduce the immunofibrotic features of the disease. Specifically, sera derived from either a-HBV or cir-HBV patients can activate healthy neutrophils, leading them to release NETs. Considering this in vitro experimental approach, we can propose that the production of NETs—at these stages of the disease—could be enhanced by the presence of soluble inflammatory proteins, namely, CCL2, IL-18, and IL-33.

In contrast, NET structures are absent in the chronic phase of the disease. In fact, the patients included in the chr-HBV group were under frequent clinical follow-up, to record the progression of the disease and the response to treatment. They showed treatment-induced stabilization, as evidenced by improvement in clinical markers and reduction in inflammation scores. Similarly, they were assessed with a low probability of progression to severe clinical disease. These clinical observations can also explain our present findings, namely, the absence of NETs and inflammatory proteins in their circulation. Consequently, serum samples from patients with chronic infection (chr-HBV) were not included in the experiments aimed at determining the protein load of NETs or the effect of NETs on human fibroblasts. On the other hand, patients with chronic infection are often at high risk of developing cirrhosis and complications of end-stage liver disease [68]. Studies have indicated that neutrophils may be inappropriately activated or become dysfunctional during liver cirrhosis, worsening liver function [69]. Markedly, IL-17-expressing neutrophils have been identified as activators of HSCs, enhancing fibrotic events [70]. Further, neutrophils can respond through NETs, in cases of compensated and decompensated cirrhosis [71,72,73].

Moreover, liver fibrogenesis is mediated by several pro-inflammatory agents, including IL-1b. Patients with cirrhosis as well as patients with chronic liver diseases are characterized by elevated serum IL-1b levels [74,75]. There are also findings demonstrating that IL-1 mediates the transition from liver injury to early liver fibrosis [76]. Our data demonstrates that citrullinated H3, a potent indicator of NETs, is elevated in the circulation of cir-HBV patients. In addition, they show that neutrophils from healthy subjects can overexpress IL-1b on mRNA level once exposed to the disease microenvironment. IL-1b is delivered and signals via NETs, a process that also occurs in several inflammatory conditions. Indicatively, IL-1b-coated NETs have been detected in both autoinflammatory diseases, such as familial mediterranean fever (FMF), and common diseases, such as gout [43,77,78], and their presence influences the immunopathology of these disorders. Hence, these findings suggest neutrophils/NETs as a potential source of IL-1b in HBV-mediated cirrhosis. The formation and progression of liver fibrosis is also based on structural cells, such as HSCs and fibroblasts. Fibroblasts respond to inflammatory stimuli and, subsequently, they can differentiate into myofibroblasts, secrete extracellular matrix proteins, and create a fibrous scar [79]. Our previous studies have focused on the remarkable interactions between NETs and fibroblasts, which can boost fibrotic responses [25,30,31]. In this study, we observe that disease NETs coated with IL-1b can increase the activation levels of fibroblasts as well as induce their fibrotic phenotype. As a result of this “close” communication, fibroblasts release collagen and display enhanced migratory capacity and, therefore, they could contribute to the progression of liver fibrosis.

Current therapies are relatively effective, having improved the quality of life and survival of infected individuals [79]. Reports have indicated, however, that the effects of available drugs are not always sustained, and as a result, individuals may develop persistent infection or other complications [80]. Hence, efforts are being made to identify new antiviral drugs as well as immunological interventions to increase the functional cure rate [79,81]. In this regard, interventions targeting the innate immune loop, e.g., against NET integrity, could be considered particularly beneficial, and their combined administration with antiviral drugs is worth investigating.

Some limitations, however, are identified in our study. Initially, there was limited access in patient biological material that did not allow for us to isolate circulating cells from HBV patients. Consequently, we only collected and stored blood sera and performed in vitro stimulations to mimic the different disease phenotypes. Similarly, we were unable to study the fibro-cirrhotic state in depth, as there was no access to biopsy material. It is known that most diagnostic centers monitor patients with non-invasive methods, resulting in limited biopsy availability. Lastly, we were unable to assess the antiviral potential of NETs, due to the degree of biosafety available. Hence, a next essential goal is to analyze whether viral remnants can be localized in extracellular structures of neutrophil origin, such as NETs.

In conclusion, this study supports innate immune mechanisms, particularly NETs, as an important piece in HBV pathophysiology. IL-1b-bearing NETs are implicated in both early and late disease stages, mediating inflammatory or/and fibrotic events. Therefore, targeting NET/IL-1b axis in combination with administration of antiviral drugs could be a promising therapeutic approach in the disease management.

## 4. Materials and Methods

### 4.1. Subjects

In this study, the following were enrolled: 11 patients with acute HBV (a-HBV), 11 patients suffering from chronic HBV (chr-HBV) infection, and 11 patients suffering from cirrhosis due to chronic HBV infection (cir-HBV). First-diagnosed a-HBV patients received no treatment prior to sampling. On the contrary, both chr-HBV and cir-HBV patients were under nucleoside analogue (NA) treatment. Chr-HBV patients were HBeAg-negative, with HBV DNA load lower than 2000 IU/mL. Inflammation scores and liver enzyme values were normal. Cir-HBV patients were HBeAg negative, with HBV DNA load higher than 2000 IU/mL. They had been diagnosed with cirrhosis within the last 6 months, considering established clinical, biochemical, and imaging tools. They had relatively low platelet count (142 ± 68.8 × 10^9^/L), increased inflammatory markers (e.g., C-reactive protein over 14 mg/L), as well as increased values of liver enzymes, including Aspartate Transaminase (AST) and Alanine Aminotransaminase (ALT). Fibrosis-4 (FIB-4) index was also calculated to assess the presence of liver fibrosis in these patients. Comorbidities like hepatocellular cancer (HCC) as well as coinfections with hepatitis D virus, hepatitis C virus, or human immunodeficiency virus were excluded (Table 1). Eleven healthy age- and sex-matched individuals (healthy individuals: HIs) served as controls. Patients’ venipuncture, clinical screening, and monitoring were performed by authorized clinicians at the University General Hospital of Alexandroupolis, in accordance with current guidelines. Subjects enrolled in this study provided informed consent. Study design complied with the Helsinki Declaration and had the approval of the Ethics Committee of the University General Hospital of Alexandroupolis, Greece (Ref. Number 12970/15-03-2023). Designated routine blood tests were performed in all subjects enrolled in this study (Table 1). Quantification of hepatitis B surface antigen (HBsAg) was performed using a microparticle immunochemical luminescence (CMIA) method (Alinity, Abbott, IL, USA).

### 4.2. Serum Collection

To isolate serum, venous blood was collected in appropriate blood collection tubes (BD Vacutainer^®^ SST II Advance Tubes, Becton, Dickinson and Company, Franklin Lakes, NJ, USA). Tubes were centrifuged at 2000× *g* for 10 min, according to the manufacturer’s instructions. Serum samples were stored at −80 °C until further analysis [25,30].

### 4.3. Neutrophil Isolation

Peripheral blood from healthy individuals (HIs) was collected in heparinized blood collection tubes (BD Vacutainer^®^ Heparin Tubes, Becton, Dickinson and Company, Franklin Lakes, NJ, USA) and HI neutrophils were isolated by Histopaque double-gradient density centrifugation (11191 and 10771, Sigma-Aldrich, St Louis, MO, USA), according to the manufacturer’s instructions. In brief, peripheral blood was centrifuged at 700× *g* at 20–25 °C for 30 min and isolated neutrophils were washed once with 1x Dulbecco’s phosphate-buffered saline (1× PBS) (Biosera, Cholet, France) (200× *g*, 10 min, 20–25 °C). Cell purity of isolated neutrophils exceeded 98%, as assessed by microscopy (May-Grünwald Giemsa staining, MGG) and/or flow cytometry.

### 4.4. Fibroblast Cell Culture

Human primary fibroblasts (Fbs) (C-12360, PromoCell, Heidelberg, Germany) were cultured at 37 °C with 5% CO_2_ in low glucose Dulbecco’s Modified Eagle Medium (DMEM, PAN Biotech, Aidenbach, Germany), supplemented with 10% *v*/*v* Fetal Bovine Serum (FBS, Capricorn Scientific, Ebsdorfergrund, Germany), 100 U/mL Antibiotic-Antimycotic solution (Biosera, Cholet, France), and 5% *v*/*v* MEM Non-Essential Amino Acids Solution (Thermo Fisher Scientific, Waltham, MA, USA). When cell culture confluency reached 80–85%, Fbs were sub-cultivated, using 0.05% Trypsin/EDTA solution (Capricorn Scientific, Ebsdorfergrund, Germany). For all experiments of this study, Fbs from passages 4–8 were used [25,31].

### 4.5. Stimulation and Inhibition Studies

#### 4.5.1. Neutrophils

HI neutrophils were cultured at 37 °C with 5% CO_2_ in Roswell Park Memorial Institute (RPMI) medium (Capricorn Scientific, Ebsdorfergrund, Germany), supplemented with 2% *v*/*v* heterologous healthy donor serum. To investigate the role of neutrophils in the disease, HI neutrophils were stimulated in vitro with the disease microenvironment, i.e., 5% *v*/*v* serum from HBV patients. In all in vitro experiments, HI neutrophils were cultured for 60 min or 3 h (37 °C, 5% CO_2_) to assess gene expression or NET structure release, respectively [25,26]. HI neutrophils that were not stimulated with patient serum served as control in all in vitro neutrophil stimulation studies.

#### 4.5.2. Fbs

To investigate the crosstalk between neutrophils and fibroblasts, Fbs were cultured in the presence of in vitro-generated NET structures (DNA concentration: 0.5 µg/mL) [30]. To dismantle the NET scaffold, NETs were pre-incubated with 1 U/mL recombinant DNase I (Takara Bio, Shiga, Japan). To hinder IL-1b signaling, Fbs were treated with Anakinra, a human IL-1 receptor antagonist (100 ng/mL; Kineret, Swedish Orphan Biovitrum AB), prior to their stimulation with NET structures. Both inhibitions were performed for 30 min at 37 °C with 5% CO_2_ [31,82]. Fbs were cultured for 3, 18, or 24 h to study gene expression, migratory/wound healing capacity, or collagen secretion, respectively [25,26,30]. Unstimulated Fbs were used as negative control in all studies.

The concentrations and time points used in both neutrophil and Fbs studies were optimized before all experiments. All substances used in these studies were endotoxin-free, as determined by a Limulus amebocyte lysate assay (Thermo Fisher Scientific, Waltham, MA, USA).

### 4.6. NET Structures Generation and Isolation

For in vitro NET structures generation 2 × 10^6^ HI neutrophils were cultured in a 6-well cell culture plate (SPL Life Sciences, Kyonggi-do, Republic of Korea) in RPMI, supplemented with 2% *v*/*v* heterologous healthy donor serum, and stimulated with serum from patients with HBV. Following a 3-hour stimulation (37 °C, 5% CO_2_), the cell culture medium was removed, cells were washed once with pre-warmed RPMI, and fresh pre-warmed RPMI was added. The cell culture plate was vigorously agitated and the cell culture supernatant was centrifuged at 20× *g* for 5 min. NET structures (NETs) were isolated from the supernatant phase [25,30,83]. HI neutrophils that were not stimulated with patient serum served as control (control NETs).

### 4.7. Immunofluorescence

HI neutrophils were seeded on poly-L-lysine-coated glass coverslips (Biocoat, NY, USA) in a 24-well cell culture plate (SPL Life Sciences, Kyonggi-do, Republic of Korea) and cultured for 3 h (37 °C, 5% CO_2_) to evaluate their NET release capacity and examine the NET protein profile. Cells were fixed with 10% formaldehyde solution (Biognost, Zagreb, Croatia) for 30 min at 4 °C and nonspecific binding sites were blocked with 6% normal goat serum (Thermo Fisher Scientific, Waltham, MA, USA) in 1x PBS (blocking solution). Following, samples were stained with a primary antibody solution, consisting of an anti-human neutrophil elastase (NE) monoclonal antibody (mAb) (1:250 dilution, Abcam, Cambridge, UK), an anti-citrullinated histone H3 (CitH3) mAb (1:200 dilution, Abcam, Cambridge, UK), or an anti-human interleukin(IL)-1b mAb (1:200 dilution, R&D System, Minneapolis, MN, USA) in blocking solution, for 1 h at room temperature (RT). A polyclonal anti-rabbit IgG AlexaFluor594 antibody (Invitrogen, Waltham, MA, USA) or a polyclonal anti-mouse IgG AlexaFluor488 antibody (Invitrogen, Waltham, MA, USA) was utilized as secondary antibody and cells were incubated in secondary antibody solution according to the manufacturer’s instructions. Finally, samples were incubated in DAPI solution (Sigma-Aldrich, St Louis, MO, USA) and mounted on microscope slides (Knittel Glass, Braunschweig, Germany) using a hardening mounting medium (Thermo Fisher Scientific, Waltham, MA, USA) [25,26,30].

Sample visualization was performed on a Nikon ECLIPSE Ti2 Inverted Microscope (Nikon, Melville, NY, USA) with a 40× oil lens (1.30 NA). Images were acquired using NIS-Elements software (Nikon, Melville, NY, USA) and analyzed in Fiji software version 2.9.0 [84].

### 4.8. Bead-Based Multiplex Immunoassay

The serum levels of C-C motif chemokine ligand 2 (CCL2), interleukin(IL)-18, and interleukin(IL)-33 were assessed using the LEGENDplexTM Multi-Analyte Flow Assay Kit (Biolegend, San Diego, CA, USA) [85]. The samples were prepared in accordance with manufacturer’s instructions and data acquisition was performed in a BD FACSAriaTM III Cell Sorter (Becton, Dickinson and Company, Franklin Lakes, NJ, USA).

### 4.9. Citrullinated Histone H3 ELISA

Citrullinated histone H3 (CitH3) was measured in both serum samples and in vitro-generated NETs, following manufacturer’s instructions (Cayman Chemical, Ann Arbor, MΙ, USA).

### 4.10. Interleukin-1b ELISA

Interleukin(IL)-1b ELISA (R&D Systems, Minneapolis, MN, USA) was performed, in line with manufacturer’s guidelines, to determine IL-1b concentration in in vitro-generated NET structures.

### 4.11. RNA Isolation, cDNA Synthesis, and Quantitative Real-Time Polymerase Chain Reaction (RT-qPCR)

RNA isolation, cDNA synthesis, and RT-qPCR were accomplished, as previously described. In brief, RNA was isolated using TRIzol Reagent (Thermo Fisher Scientific, Waltham, MA, USA), cDNA was synthesized with a commercially available kit (Takara Bio, Shiga, Japan), and RT-q PCR was performed using an appropriate SYBR green RT-qPCR master mix (Kapa Biosystems, Wilmington, MA, USA). The expression of IL-1b and Actin Alpha 2 Smooth Muscle (ACTA2) were examined in neutrophils and Fbs, respectively. Normalization of gene expression was achieved with Glyceraldehyde 3-phosphate dehydrogenase (GAPDH) following the housekeeping gene normalization method. All RT-qPCR primers were designed using Beacon Designer software version 4.0. Details regarding primers and RT-qPCR conditions are provided in Supplemental Table S1. For RT-qPCR data analysis, the 2^−ΔΔCt^ method was applied [25,29,30,31].

### 4.12. Migration/Wound Healing Assay

To assess the migratory/healing capacity of Fbs, cells were seeded in 24-well cell culture plate (SPL Life Sciences, Kyonggi-do, Republic of Korea) and a wound was created using an appropriate scratcher tip (SPL Life Sciences, Kyonggi-do, Republic of Korea) when cell confluency reached 90%. Then, stimulation and inhibition studies were performed. After 20 h of incubation, the migratory/wound healing potential of Fbs was assessed by MGG stain [25,30,86].

### 4.13. May–Grünwald Giemsa (MGG) Stain

To examine the migration/wound healing capacity of Fbs, MGG stain was performed. To start with, cells were stained with May–Grünwald stain for 5 min at RT. Following a washing step with water, Fbs were incubated with Giemsa stain (dilution 1:10) for 20 min at RT. Finally, samples were washed with water and let to air-dry [25,30]. Sample visualization was achieved with an OLYMPUS Upright Microscope (OLYMPUS Corporation, Tokyo, Japan), equipped with a SC30 digital camera (OLYMPUS Corporation, Tokyo, Japan), with a 4× air lens (0.10 NA) (PLCN4X/0.1, OLYMPUS Corporation, Tokyo, Japan). OLYMPUS cellSens Entry software (OLYMPUS Corporation, Tokyo, Japan) was used for image acquisition. Final images were produced with Fiji software version 2.9.0 [84].

### 4.14. Collagen Assay

To assess collagen release, Fbs were seeded in 6-well cell culture plates (SPL Life Sciences, Kyonggi-do, Republic of Korea) and stimulation and inhibition studies were performed for 24 h (37 °C, 5% CO_2_). Then, cell culture supernatants were collected, and soluble collagen (type I-V) concentration was determined by Sircol Soluble Collagen Assay kit (Biocolor Life Science Assays, Co Antrim, UK) [30]. The assay was performed in accordance with the manufacturer’s instructions and recommendations.

### 4.15. Statistical Analysis

Statistical analyses were performed using GraphPad Prism software version 8.4.2 (GraphPad Software, Inc., San Diego, CA, USA). For comparisons among more than 2 non-paired groups, the Kruskal–Wallis test, followed by Dunn’s multiple comparison test, was used. Comparisons among more than 2 paired groups were performed with the Friedman test, followed by Dunn’s multiple comparison test. Spearman’s rank correlation coefficients test at 95% confidence intervals (CIs) was utilized for bivariate correlation analysis. Statistical significance was set to *p* < 0.05. All data are presented as mean ± standard deviation (SD).

## Figures and Tables

**Table 1:** Baseline characteristics of study population.

	Acute HBV	Chronic HBV	Early Cirrhosis	Control
Age	55 ± 9.8	56.21 ± 9.4	69.1 ± 11.9	55 ± 9.8
Male (%)	7 (63.6%)	8 (72.7%)	8 (72.7%)	9 (81.8%)
Laboratory findings				
WBC (10^9^/L)	5.1 ± 1.3	5.7 ± 2.6	6.6 ± 3.2	5.3 ± 5.6
Hg (g/L)	13.4 ± 1.3	13.8 ± 0.4	13.4 ± 2	14.3 ± 1.3
PLT (10^9^/L)	162 ± 50.4	146 ± 48.1	142 ± 68.8	241 ± 43.7
SGOT (Mean ± SD)	409.5 ± 498.9	30 ± 4.2	29.3 ± 7.2	15.6 ± 9.5
SGPT (Mean ± SD)	271.8 ± 404.5	34.2 ± 4.4	30.3 ± 22.5	13.8 ± 9.5
gGT (Mean ± SD)	145.7 ± 83.5	98.2 ± 36.2	56.6 ± 102.2	12.2 ± 3.8
HBsAg	3161.4 ± 1618.3	1806.7 ± 1025.4	3850.5 ± 2080.3	0.03 ± 0.03

## Data Availability

Data is contained within the article or Supplementary Material.

## Supplementary Materials

The following supporting information can be downloaded at: https://www.mdpi.com/article/10.3390/ijms26125733/s1.
