# Supplementary material for: IL-1b-Bearing NETs: Bridging Inflammation to Early Cirrhosis in Hepatitis B
Source: Int J Mol Sci. 2025 Jun 15;26(12):5733. doi: 10.3390/ijms26125733 (PMC12193664; doi:10.3390/ijms26125733)
Supplement: Supplementary file 1 [file ijms-26-05733-s001.zip › Figure S1.pdf]

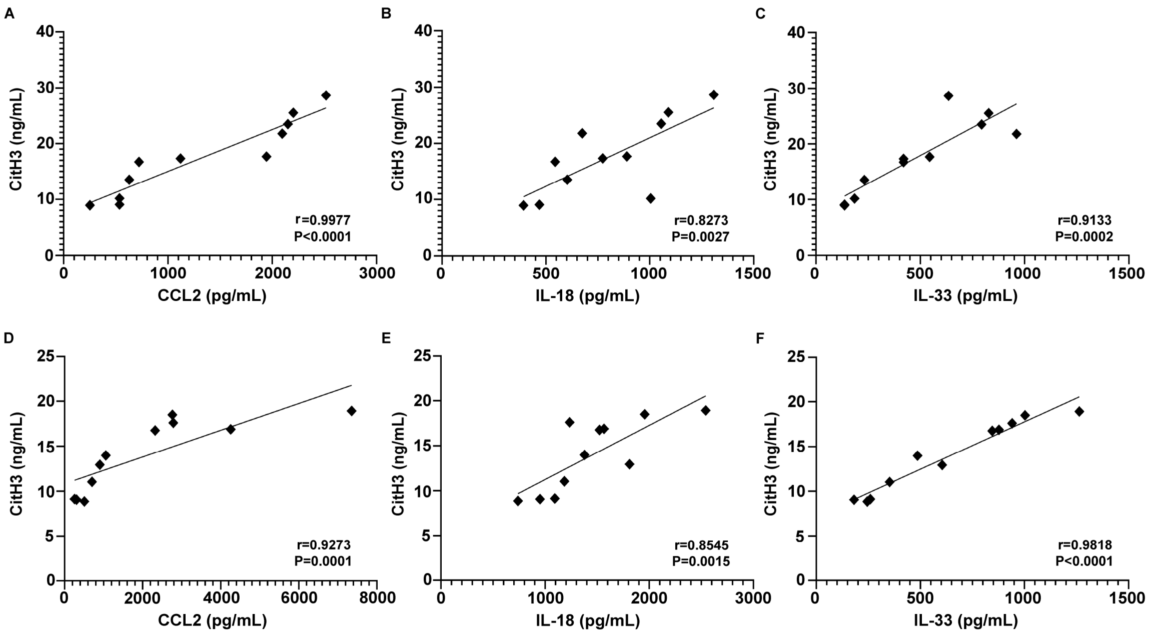

Figure S1. Correlation between soluble inflammatory cytokines and NETs. Correlations between CitH3 and (**A**) CCL2, (**B**) IL-18 and (**C**) IL-33 levels in the serum of a-HBV patients (n=11 subjects). Correlations between CitH3 and (**D**) CCL2, (**E**) IL-18 and (**F**) IL-33 levels in the serum of cir-HBV patients (n=11 subjects). Spearman's r and P values are shown. Statistically significant: $P < 0.05$. a-HBV: acute HBV; cir-HBV: early cirrhotic stage.
