# Supplementary material for: IL-1b-Bearing NETs: Bridging Inflammation to Early Cirrhosis in Hepatitis B
Source: Int J Mol Sci. 2025 Jun 15;26(12):5733. doi: 10.3390/ijms26125733 (PMC12193664; doi:10.3390/ijms26125733)
Supplement: Supplementary file 1 [file ijms-26-05733-s001.zip › Supplementary Table S1.pdf]

Table S1. Sequence of primers and RT-qPCR conditions

Gene	Primer	Primer Sequence	RT-qPCR conditions
IL-1b	FRD	5' TTACAGTGGCAATGAGGATGAC 3'	1. 52ºC for 5 min<br>2. 95 ºC for 2 min<br>3. 35 cycles of:<br>I. 95 ºC for 15 sec<br>II. 56ºC for 40 sec<br>4. 52 ºC for 5 min<br>5. Melting curve analysis
	REV	5' AGTGGTGGTCGGAGATTCG 3'	
ACTA2	FRD	5' ACGCACAACTGGCATCG 3'	
	REV	5' CGGACAATCTCACGCTCAG 3'	
GAPDH	FRD	5' AGGTGGTCTCCTCTGACTTC 3'	
	REV	5' CTGTTGCTGTAGCCAAATTCG 3'	
FRD: forward primer, REV: reverse primer			
